# Complete sequence of chinese wild *Vitis davidii* chloroplast DNA

**DOI:** 10.1080/23802359.2018.1508379

**Published:** 2018-10-26

**Authors:** Ningbo Zhang, Wei Shen, Rui Wang, Junxiang Zhang

**Affiliations:** a School of Agronomy, Ningxia University, Yinchuan, P. R. China;; b Ningxia Engineering and Technology Research Center of Grape and Wine, Ningxia University, Yinchuan, P. R. China;; c Engineering Research Center of Grape and Wine, Ministry of Education, Ningxia University, Yinchuan, P. R. China;; d School of Vine and Wine, Ningxia University, Yinchuan, P. R. China

**Keywords:** Wild-growing, Vitis davidii, Chloroplast genome, phylogeny

## Abstract

*Vitis davidii* Foёx is an important wild grape species with extremely high diseases resistance, health promoting properties, and utilization value. Here, the whole-genome high-throughput sequencing data was mined to determine the entire chloroplast genome of this *Vitis* species. This circular DNA is 161,065 bp in size, counting a pair of inverted repeats (24,802 bp each). The chloroplast genome consists of 125 genes, including 86 protein-coding genes, 31 transfer RNAs, and 8 ribosomal RNAs. A phylogenetic analysis based on the complete chloroplast genome sequences in plants showed that *V. davidii* formed a different clade from other four congeneric species of the family *Vitaceae*. This complete chloroplast genome will provide valuable information for future evaluation, conservation, and utilization of *V. davidii.*

Spine Grape *(Vitis davidii* Foёx), is one of striking wild germplasms of the East Asian *Vitis* spp., which is predominantly distributed in South China (Meng et al. 2012). This geographical distribution of this species confers their strong tolerance to moisture and heat (Meng et al. 2012; Xu et al. 2014). Also, this species displays extremely high resistance to *Sphaceloma ampelinum* de Bary, *Coniathyrium diplodilla* (Speg.) Sacc.), and *Glomerella cigulata* (ston.) Spault et Schrenk. Although it is a wild-growing *Vitis* species, several vines of this species were found to have prominent bisexual flowers. At present, spine grapes were mainly made available as a fresh edible fruit and a small part has also been used for making wines in China. However, it has not yet been effectively developed and fully utilized.

As one of the important genetic marker sources, chloroplast genomes are widely used for phylogenetic analyses, genetic diversity evaluation, and plant molecular identification (Dong et al. 2014; Li et al. 2016). There are only four chloroplast genomes of the genus *Vitis* annotated, including *V. vinifera* (Jansen et al. 2006), *V. amurensis* (Xie et al. 2016), *V. aestivalis*, and *V. rotundifolia*. In this study, we determined the complete chloroplast genome sequence of *V. davidii* (GenBank accession no: MG251741), hence adding a new reported chloroplast genome to the genus *Vitis*. Young and healthy leaves were sampled from *V. davidii* Foёx in the germplasm nursery (N38°30'43.44", E106°08'19.67") of Ningxia University (Ningxia, China). Genomic DNAs were extracted, quantified and further sequenced on the Illumina Hiseq Xten Platform (Illumina, San Diego, CA). The living material and DNA were stored in Ningxia University, China. The phylogenetic analysis was carried out with the 15 reported plant chloroplast genome sequences using PhyML (Stéphane et al. 2005).

The complete chloroplast genome of *V. davidii* is 161,065 bp long and consists of two inverted repeated regions (IRa and IRb) of 24,802 bp. The chloroplast genome of *V. davidii* consists of 125 genes, including 86 protein-coding genes, 31 transfer RNAs, and 8 ribosomal RNAs. Intron-exon structure analysis indicated the majority (115 genes, 92%) are genes with no introns, whereas 8 (6.4%) genes contain a single intron and 2 protein-coding genes harbour two introns. The genome organization, and the relative positions of 125 individual genes in chloroplast genome of *V. davidii* were highly similar to those of the *V. vinifera* (Number: 84/37/8) (Jaillon et al. 2007) and those of the *V. amurensis* (Number: 88/37/8) (Xie et al. 2016) except for some minor differences in numbers of genes.

The phylogenetic analysis showed five *Vitis* species (*V. vinifera*, *V. aestivalis*, *V. amurensis*, *V. rotundifolia*, and *V. davidii*), *Ampelopsis glaandulosa* and a *Tetrastigma hemsleyaanum* were clustered together with a well-supported cluster, where *V. davidii* formed a different clade from other four congeneric species of the family *Vitaceae* (Figure 1). This suggests that *V. davidii* is a sister group of the other *Vitis* species, that split before the divergence among *V. vinifera*, *V. aestivalis*, *V. amurensis*, and *V. rotundifolia*.

**Figure 1.**
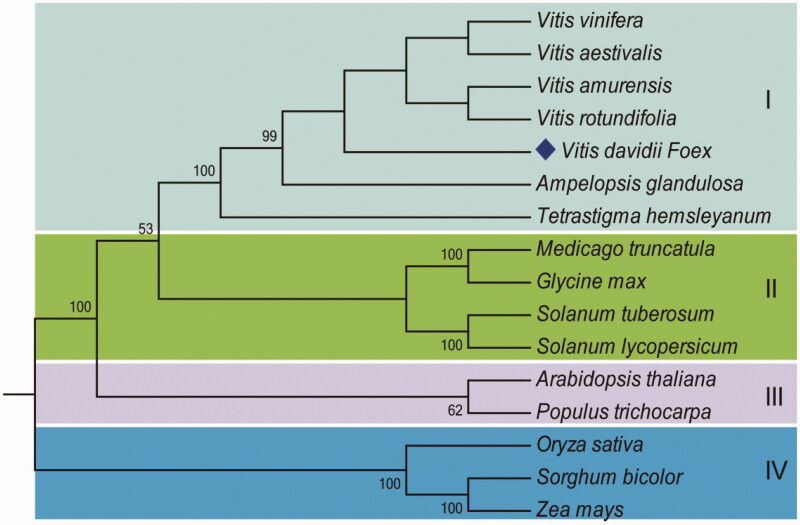
Phylogenetic tree of 16 complete chloroplast genome sequences shows the 4 distinct clusters. GenBank accession numbers: *Vitis vinifera* (NC_007957), *Vitis davidii* (MG251741, in this study), *Vitis amurensis* (KX499471), *Vitis aestivalis* (KT997470), *Vitis rotundifolia* (KF976463), *Ampelopsis glandulosa* (KT831767), *Tetrastigma hemsleyanum* (KT033563), *Sorghum bicolor* (NC_008602), *Zea mays* (NC_001666), *Oryza sativa* (NC_001320), *Medicago truncatula* (NC_003119), *Glycine max* (NC_007942), *Solanum tuberosum* (NC_008096), *Solanum lycopersicum* (NC_007898), *Arabidopsis thaliana* (NC_000932), and *Populus trichocarpa* (NC_009143).
